# Ethanol induces upregulation of the nerve growth factor receptor CD271 in human melanoma cells via nuclear factor-κB activation

**DOI:** 10.3892/ol.2015.3343

**Published:** 2015-06-09

**Authors:** GERMANA RAPPA, FABIO ANZANELLO, AURELIO LORICO

**Affiliations:** Cancer Research Center, Roseman University College of Medicine, Las Vegas, NV 89135, USA

**Keywords:** CD271, melanoma, nuclear factor-κB

## Abstract

Alcohol consumption is one of the most important, and potentially avoidable, risk factors of human cancer, accounting for 3.6% of all types of cancer worldwide. In a recent meta-analysis, a 20% increased risk of melanoma was linked with regular alcohol consumption. In the present study, the effect of ethanol exposure on the expression of the nerve growth factor receptor, CD271, in human FEMX-I melanoma cells was investigated. Consistent with the derivation of melanocytes from the neural crest, the majority of melanomas express CD271, a protein that is crucial for maintaining the melanoma stem cell properties, including the capacity of self-renewal and resistance to chemotherapy and radiotherapy. Analysis of CD271-sorted subpopulations and clones of FEMX-I cells indicated no hierarchical organization of CD271^+^ and CD271^−^ cells. In addition, CD271 expression was lost upon growth of FEMX-I melanoma cells in cancer stem cell-like conditions, while it was greatly increased upon CD133 knockdown or exposure to ethanol. After 24-h exposure to 100, 200 and 400 mM ethanol, the percentage of CD271^+^ cells increased from 14% in control cells to 24, 35 and 88%, respectively. An increase in the percentage of CD271^+^ cells was already evident 8 h after ethanol exposure and reached a maximum at 48 h. Ethanol-induced upregulation of CD271 was mediated by nuclear factor-κB (NF-κB). In fact, exposure of FEMX-I cells to 100–400 mM ethanol for 24 h resulted in a concentration- and time-dependent increase in NF-κB activity, up to 900% that of control cells. NF-κB activation was due to a decrease in p50 homodimers, which occupy the NF-κB binding site, blocking transactivation. No effects of ethanol on 9 additional signaling pathways of FEMX-I cells were observed. In the presence of CD271 blocking antibodies, NF-κB activation was not prevented, indicating that ethanol did not target CD271 directly. These data demonstrate that ethanol induces expression of CD271 in FEMX-I cells via NF-κB activation and indicate a possible molecular link between ethanol exposure and melanoma formation.

## Introduction

Malignant melanoma (MM) accounts for ~5% of all the newly diagnosed cases of cancer in the USA (1). Alcohol consumption is one of the most important, and potentially avoidable, risk factors of human cancer (2). Approximately 3.6% of all types of cancer (5.2% in men; 1.7% in women) are attributable to alcohol consumption worldwide (3). In addition, exposure to ultraviolet radiation from the sun, particularly intermittent and exposure resulting to sunburn, is the main established cause of MM (4,5). However, in a systematic review and meta-analysis of published data, which was based on 16 studies and a total of 6251 cases of cutaneous melanoma, a 20% increased risk was identified for individuals who regularly consume alcohol compared with individuals with no consumption or occasional drinking (6).

The nerve growth factor receptor, CD271, is a protein expressed in the vast majority of human melanomas (7,8) and in particular in melanoma stem cells (9,10). CD271 was recently recognized as a crucial molecule that drives melanoma initiation and metastasis by a mechanism that is currently unknown (9,10), thus endowing melanoma cells with stem-like properties. In addition to forming a high-affinity binding site when co-expressed with trk receptors (11), CD271 may also function autonomously in order to activate signaling cascades involved in apoptosis (12). Nerve growth factor and neurotrophin-3, which are neurotrophins and CD271 ligands, are potent chemotactic agents for human melanoma cells that express CD271 (13). The sustained expression of CD271 during melanocyte development may support the process of melanoma formation (4). Previous studies have also demonstrated that the stem-like features of melanoma cells are associated with the presence of CD133 (also known as prominin-1), which is a glioma and neural stem cell marker (6,7). The group of the current study has previously reported that CD133 expression is correlated with the high tumorigenic and metastatic potential of melanoma cells, and facilitates cell motility (14).

Due to its restricted expression profile, with high expression in melanoma and low expression in healthy tissues (besides the neural tissue), CD271 is an ideal therapeutic target. Therefore, it is important to investigate its function(s) and the underlying mechanisms that regulate its expression in MM. In the present study, human FEMX-I melanoma cells, which were originally derived from lymph node metastasis tissues of a melanoma patient (15), were employed in order to investigate the effects of ethanol exposure on the expression of CD271.

## Materials and methods

### 

#### Cell culture

The human FEMX-I cell line was originally derived from a lymph node metastasis of a patient with MM and obtained from Dr Øystein Fodstad (Oslo University Hospital HF, Oslo, Norway) (15). Cells were routinely cultured in RPMI-1640 medium (Mediatech, Inc., Manassas, VA, USA; http://www.cellgro.com) supplemented with 10% fetal bovine serum (Atlanta Biologicals, Lawrenceville, GA, USA; http://www.atlantabio.com) at 37°C in a 5% CO_2_ humidified incubator, using 3–15 passages. Subsequently, the cells were stored in aliquots in liquid nitrogen and kept in culture for <3 months. The complete culture medium for FEMX-I cells cultured as spheroids consisted of α-minimum essential medium (Gibco Life Technologies, Grand Island, NY, USA), B27® supplement (Gibco), 100 units/ml penicillin, 100 µg/ml streptomycin and 2 mM L-glutamine. Continuous exposure of 50,000 FEMX-I cells/ml to 100–400 mM ethanol in complete medium for up to 48 h was performed in sealed tissue culture plates to minimize evaporation, and HEPES (pH 7.4; EMD Millipore, Billerica, MA, USA) was employed to maintain the pH. The cells were routinely tested for mycoplasma contamination using the Venor GeM mycoplasma detection kit (Sigma-Aldrich, St. Louis, MO, USA) and DAPI staining, while the samples were authenticated by a morphology check every 2 weeks.

#### CD133 downregulation

Generation of CD133-knockdown FEMX-I /SUPER^773^ cells was previously described (14). Briefly, the vector pSUPER.retro.neo + GFP (pSUPER) from OligoEngine (Seattle, WA, USA; http://www.oligoengine.com) was used to generate a retroviral plasmid expressing short hairpin RNA, based on the cDNA of CD133 (SUPER^773^), corresponding to nucleotides 773–792 (GACCCAACATCATCCCTGT; Genbank accession no. NM006017). FEMX-I cells were transduced with the GALV-pseudotyped retroviral vectors SUPER^773^. Transduced cells were subsequently sorted based on GFP fluorescence.

#### Flow cytometric analysis

Analysis of CD271 expression was performed by a phycoerythrin-conjugated mouse monoclonal anti-CD271 antibody (clone C40-1457; catalog no. 557196; BD Biosciences, Franklin Lanes, NJ, USA) diluted 1:20 in PBS + 0.5% bovine serum albumin using an iCyt Reflection flow cytometer (iCyt, Champaign, IL, USA).

#### Lentiviral vector transduction

Cells were transduced with VSV-pseudotyped lentiviral particles expressing firefly luciferase under the control of different promoters, (TCF-LEF, RBP-Jk, p53, SMAD2/3/4, E2F/DP1, Myc/Max, HIF1A, Elk-1/SRF and AP-1) and expressing the puromycin N-acetyl-transferase gene under the control of hPGK promoter (pCignal/luc; SABiosciences, Frederick, MD, USA). For the transduction, the RetroNectin method was used as follows: Retroviral supernatants were preloaded onto plates coated with recombinant fibronectin (RetroNectin®; Takara Bio Inc., Otsu, Japan) and centrifuged at 950 × g for 30 min at 4°C. This process was repeated with fresh supernatant. Next, the supernatant was removed and the plates washed with phosphate-buffered saline (PBS) prior to the addition of cells. Transduced cells were isolated via selection with 2 g/ml puromycin (EMD Millipore).

#### Luciferase assay

Cell extracts were prepared and luciferase activity was measured using the One-Glo Luciferase Reporter Assay System, according to the manufacturer's instructions (Promega Corporation, Madison, WI, USA). The luciferase activity assays were performed with a 20/20 luminometer (Turner BioSystems, Sunnyvale, CA, USA). To investigate the effect of CD271 blocking antibodies on ethanol-induced nuclear factor-κB (NF-κB) activation, cells were pre-incubated with 10 µg/ml monoclonal mouse anti-human CD271 antibody [clone ME20.4 (catalog no. 345102; BioLegend, Inc., San Diego, CA, USA) or clone C40-1457 (BD Biosciences)] for 1 h prior to addition of ethanol.

#### Confocal microscopy

Cells (50,000/chamber) were seeded on chamber slides coated with poly-L-lysine, and grown overnight. Following aspiration of media, the cells were fixed in 4% paraformaldehyde, washed with PBS, permeabilized in 0.5% Tween 20 and blocked with goat serum (Rockland Inc., Gilbertsville, PA, USA). Subsequent to washing with PBS, the cells were incubated overnight at 4°C with 5 µg/ml mouse anti-CD271 monoclonal antibody (clone ME20.4) in PBS + 0.5% bovine serum albumin, followed by two further washes, and a 45 min incubation at room temperature with a 0.5 µg/ml phycoerythrin-conjugated polyclonal goat anti-mouse IgG F(ab')2 antibody (catalog no. 710–1831; Rockland Immunochemicals, Limerick, PA, USA). Cells were imaged using confocal laser-scanning microscopy on a Nikon A1R+ (Nikon Corporation, Melville, NY, USA) using a galvano or resonant scanner and a 60X Apo total internal reflection fluorescence (TIRF) oil-immersion objective. Images were captured using NIS-Elements software (Nikon Corporation) and the raw images were processed using Fiji (http://www.fiji.sc/Fiji) (16).

#### TIRF microscopy

A Nikon Eclipse Ti inverted microscope equipped with a Nikon TI-TIRF Illuminator unit (Nikon Corporation) and a 60X Apo-TIRF oil-immersion objective (numerical aperture, 1.49) were used to capture images. The cells were kept in a humid, live-cell chamber set at 37°C and 5% CO_2_ throughout the experiment. Images were recorded using a Nikon Digital Sight DS-U3 CCD camera and processed using Nikon NIS-Elements AR 3.22 software and Fiji software. The TIRF microscope was able to excite fluorophores only ~100 nm from the coverslip.

#### NF-κB-DNA binding activity by ELISA

The NF-κB nuclear-binding activity was determined using the TransAM® NF-κB family ELISA kit (Active Motif, Rixensart, Belgium). This ELISA kit is able to detect the NF-κB subunits p65, p50, c-Rel, Rel-B and p52 (17). FEMX-I melanoma cells (2×10^6^ cells/well) were seeded in 24-well culture plates and treated with ethanol (various times and concentrations). Nuclear extracts were prepared using a nuclear extraction kit (Active Motif). Subsequently, the protein content in the nuclear extracts was quantified using a BCA Protein Assay kit (Pierce, Bonn, Germany). Equal amounts (7 g) of nuclear proteins were placed into a 96-well plate coated with oligonucleotides covering the NF-κB consensus sequence of 5′-GGGRNYYYCC-3′ (in which R is a purine, Y is a pyrimidine, and N is any nucleotide).

Binding of the different NF-κB subunits to the immobilized oligonucleotides was detected using subunit-specific antibodies (TransAM® ELISA kit), followed by immunostaining with horseradish peroxidase-labeled secondary antibodies (TransAM® ELISA kit). The chromogenic reaction was measured at 450 nm in a Synergy 2 ELISA reader (BioTek Instruments, Inc., Winooski, VT, USA). For each run, a series of positive and negative controls was performed to ensure specificity in the detection of the DNA binding activity of NF-κB.

## Results

### 

#### Expression of CD271 in FEMX-I cells

**Figure 1.**
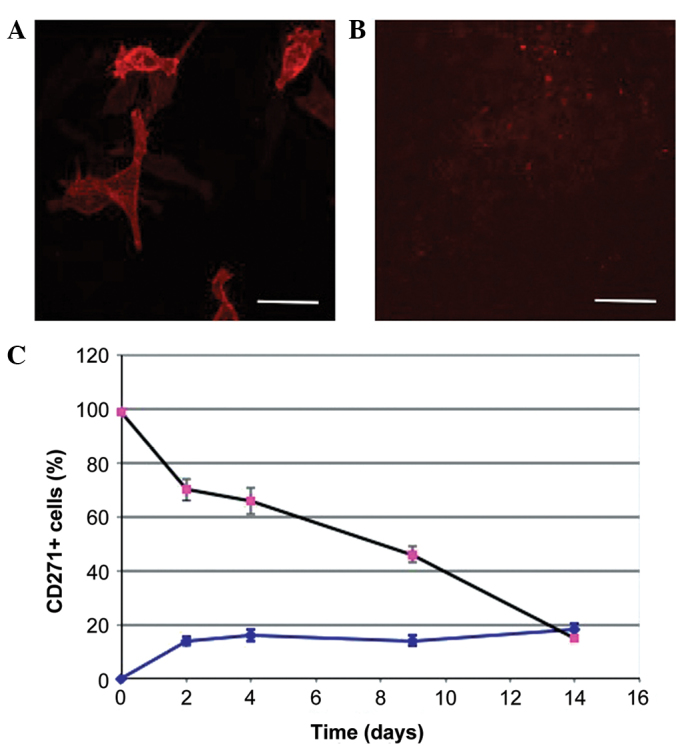
Protein expression of CD271 in FEMX-I melanoma cells. Confocal fluorescent micrographs reveal the expression of CD271 (TRITC, red) in FEMX-I cells (A) growing in serum-additioned medium or (B) upon growth as spheroids in serum-free medium and mechanical dissociation. (C) Percentage of FEMX-I cells expressing CD271 on the cell surface as measured by flow cytometry. CD271^+^ (squares) and CD271^−^ cells (circles) were sorted and plated in serum-additioned medium for 14 days. Each point represents the mean of three independent experiments. Error bars, standard deviation; scale bars, 25 mm.

The presence and localization of CD271 within FEMX-I cells was investigated by confocal fluorescence microscopy. CD271 was present in a subset of FEMX-I cells in the plasma membrane and in the cytoplasm (Fig. 1A). Flow cytometric analysis revealed that 14% of cells expressed CD271 on the cell membrane. Upon growth of FEMX-I cells as spheroids under stem cell-like conditions for 5 days, an almost complete loss of both the membrane and cytoplasmic pools of CD271 was observed (Fig. 1B). In addition, flow cytometric analysis was performed and revealed that CD133-knockdown FEMX-I cells overexpressed CD271. The percentage of CD271^+^ cells and the average level of expression per cell were higher in the CD133-knockdown FEMX-I/SUPER^773^ cell line, which is a previously developed subline with <5% residual CD133 expression (14) compared with that in parental FEMX-I cells (Table I). Furthermore, the FEMX-V sub-line, a less metastatic and aggressive subline of FEMX-I that expresses lower levels of CD133 (14,18), presented higher expression levels of CD271 (Table I).

#### Absence of hierarchical organization of CD271^+^ and CD271^−^ cells

To understand whether a hierarchy existed between CD271^+^ and CD271^−^ FEMX-I cells, 24 single-cell clones were established from the parental cell line by limiting dilution; these were then analyzed by flow cytometry after 4 weeks to determine the CD271 surface expression. Each clone was found to contain a mixture of positive and negative cells, with CD271^+^ cells ranging between 0.3 and 24.7% of the total cell population (Table II). In addition, CD271^+^ and CD271^−^ cells, which were sorted on the basis of surface fluorescence, progressively reconstituted mixed CD271^+^ and CD271^−^ cell populations. Thus, CD271^+^ sorted cells in the culture progressively decreased their positivity, while CD271^−^ sorted cells progressively reacquired CD271 expression. As a result, after 15 days in culture, the two sorted subpopulations had similar percentages of CD271^+^ cells (Fig. 1C). Notably, not all CD271^−^ cells had cytoplasmic expression of CD271, suggesting that a population of cells not expressing CD271 at all exists in FEMX-I cells. These data excluded the existence of a hierarchical organization of CD271^+^ and CD271^−^ cells. No statistically significant differences in the capacity to grow as melanospheres were observed between CD271-high and CD271-low clones (data not shown).

#### Effect of ethanol on CD271 expression

**Figure 2.**
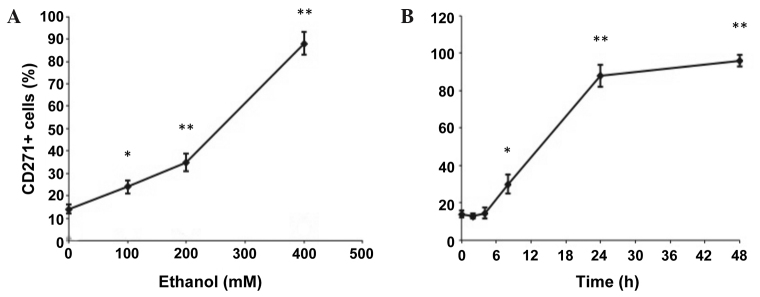
Ethanol-mediated increase in the fraction of CD271^+^ cells. FEMX-I cells were exposed to (A) different concentrations of ethanol for 24 h or (B) 400 mM ethanol for different times. CD271 positivity was measured by flow cytometry. *P<0.05 and **P<0.01 vs. no ethanol (unpaired Student's t-test). Error bars, standard deviation.

Flow cytometric analysis was used to assess whether exposure of FEMX-I cells to different concentrations of ethanol changed the percentage of CD271^+^ cells. After 24-h exposure to 100, 200 and 400 mM ethanol, the percentage of CD271^+^ cells increased from 14% in untreated cells to 24, 35 and 88%, respectively (Fig. 2A). By contrast, ethanol had no effect on the expression of CD133 in FEMX-I cells (data not shown). An increase in the percentage of CD271^+^ cells was already evident after 8 h, and reached a maximum at 48 h (Fig. 2B).

#### Association between CD271 and NF-kB

**Figure 3.**
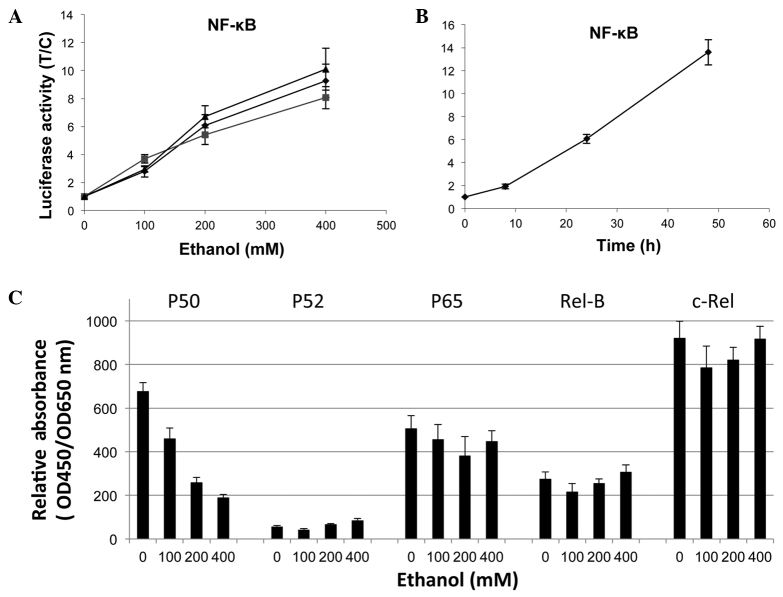
Ethanol-induced NF-κB activation in FEMX-I cells. FEMX-I cells, stably transduced with a lentiviral reporter construct that expressed firefly luciferase downstream of a specific NF-κB transcriptional response element, were exposed to (A) different concentrations of ethanol for 24 h or (B) 400 mM ethanol for 48 h. NF-κB activity was measured by a luciferase assay. At 1 h before ethanol treatment, cells were preincubated with 10 mg/ml anti-CD271 clone ME20.4 (squares), clone C40-1457 (triangles) or phosphate-buffered saline (circles). (C) Nuclear extracts of FEMX-I cells exposed to different concentrations of ethanol for 24 h were prepared and analyzed by an ELISA specific for the five subunits of NF-κB (p65, p50, c-Rel, Rel-B and p52) using the TransAM assay. Equal amounts (7 mg) of nuclear proteins were placed into a 96-well plate coated with oligonucleotides that cover the NF-κB consensus sequence. Binding of the different NF-κB subunits to the immobilized oligonucleotides was detected by using subunit-specific antibodies followed by immunostaining with horseradish peroxidase-labeled secondary antibodies. The chromogenic reaction was measured at 450 nm in an ELISA reader. For each run, a series of positive and negative controls was performed to ensure specificity of detection of NF-κB-DNA binding activity. Exposure to ethanol significantly decreased p50 levels vs. control (P<0.05 for 100 mM; P<0.005 for 200 and 400 mM). Error bars, standard deviation. NF, nuclear factor; OD, optical density.

As an interaction between members of the tumor necrosis factor receptor (TNFR) superfamily of receptors, which includes CD271, and the NF-κB signaling pathway has been reported previously (19), the present study investigated whether NF-κB mediated the effect of ethanol on CD271 expression. Using a lentiviral reporter construct that expressed firefly luciferase downstream of a specific NF-κB transcriptional response element (NF-κB-*luc*), FEMX-I cells were found to constitutively express NF-κB (5,500±600 units/10,000 cells). To investigate whether CD271 was an NF-κB target gene, FEMX-I/NF-κB-luc was exposed to 100–400 mM ethanol for 24 h and the NF-κB activity was measured. Ethanol treatment resulted in a concentration-dependent increase in NF-κB activity, up to 900% of the control cell activity (Fig. 3A). The effect of ethanol was evident already at 8 h, reaching a maximum 14-fold increase upon exposure of FEMX-I cells to 400 mM ethanol for 48 h (Fig. 3B). The effect of ethanol was specific for NF-kB. In fact, exposure to 100–400 mM ethanol for 24 h did not change the reporter activity, measured by a luciferase assay, of the following transcription factors and signaling pathways: TCF/LEF (Wnt pathway), RBP-Jk (Notch pathway), p53, SMAD2/3/4 (TGF-β pathway), E2F/DP1 (pRb pathway), Myc/Max, HIF1A (hypoxia pathway), Elk-1/SRF (MAPK/ERK pathway) and AP-1 (MAPK/JNK pathway) (data not shown). Furthermore, to determine the mechanism underlying the NF-κB activation by ethanol, FEMX-I cells exposed to different concentrations of ethanol for 24 h were investigated by an ELISA specific for the five subunits of NF-κB (including p65, p50, c-Rel, Rel-B and p52). Upon treatment of FEMX-I cells with 100–400 mM ethanol for 1 h, the p50 levels decreased proportionally to the ethanol concentration; by contrast, the levels of p65, c-Rel, Rel-B and p52 remained unchanged upon ethanol treatment (Fig. 3C). Since p50 homodimers normally occupy the binding sites under baseline conditions and block transactivation, the ethanol-induced decrease in p50 levels potentially favors the binding of other NF-κB subunits, including p50 heterodimers, and leads to transactivation.

As previous studies have reported that multiple signaling systems, including the members of the TNFR superfamily, activate NF-κB upon binding of their respective physiological ligands (20,21), the present study investigated whether the ethanol effect on NF-κB was mediated by a direct effect on CD271. Therefore, FEMX-I cells were exposed to ethanol in the presence of CD271 blocking antibodies, revealing that NF-κB activation was not prevented (Fig. 2), which indicated that ethanol did not target CD271 directly.

#### Confirmation of ethanol-induced increase in CD271+ cells by fluorescence microscopy

**Figure 4.**
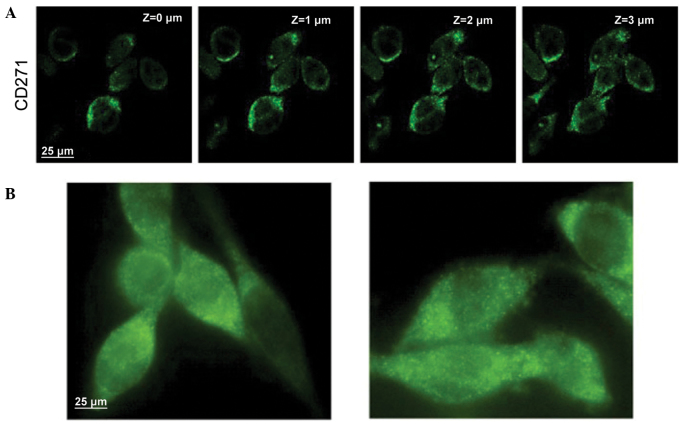
Effects of ethanol on CD271 cellular distribution. (A) Confocal fluorescence imaging of CD271 in FEMX-I cells upon exposure to 400 mM ethanol for 24 h. (B) Total internal reflection fluorescence imaging of FEMX-I cells upon exposure to 400 mM ethanol for 24 h shows extensive CD271 surface staining (green, FITC-conjugated anti-mouse secondary antibody).

The ethanol-induced increase in CD271^+^ cells was confirmed by confocal fluorescence microscopy. Plasma membrane and cytoplasmic positivity for CD271 following exposure to 400 mM ethanol for 24 h (Fig. 4A) were observed in virtually all cells. TIRF microscopy, which is capable of imaging a thin layer of the cell surface <100 nm, confirmed the extensive presence of CD271 at the membrane level subsequent to ethanol treatment (Fig. 4B).

## Discussion

The present study identified that exposure of FEMX-I melanoma cells to ethanol caused a rapid upregulation of CD271 expression via NF-κB activation. In particular, a decrease in the intracellular level of the p50 subunit of the NF-κB complex was responsible for NF-κB activation. Under baseline conditions, p50 homodimers occupy the NF-κB binding sites and block transactivation, while a decrease in p50 levels favors the binding of other NF-κB subunits, including p50 heterodimers, and leads to transactivation. Consistent with our observations, Gukovskaya *et al* (22) have previously reported that ethanol can regulate NF-κB in rat pancreatic acinar cells. The present study identified that NF-κB is constitutively activated in FEMX-I cells, which is supported by analogous findings in other human melanoma cell lines (23,24) and by the observation that NF-κB regulates a number of important biological and pathological processes in human melanoma cells (23–25). Our findings are also in agreement with a previous study reporting that ethanol increased the percentage of CD271^+^ neurons in rats (26). While other studies have demonstrated that activation of CD271 by nerve growth factor causes the translocation of NF-κB to the nucleus and promotes cell survival (27,28), CD271 was not found to activate the NF-κB signaling pathway in the FEMX-I cells. In fact, blocking CD271 with specific antibodies did not interfere with ethanol-induced activation of the NF-κB signaling pathway.

Employing single-cell cloning and following the expression of CD271 in sorted FEMX-I subpopulations, the current study observed absence of hierarchical association between CD271^+^ and CD271^−^ cells. This suggests that CD271 does not identify a melanoma stem cell subpopulation in FEMX-I cells. In addition, CD271 expression was lost upon growth in cancer stem cell-like conditions (growth as spheroids in serum-free medium). Notably, CD271 expression greatly increased upon CD133 knockdown, and flow cytometric analysis of parental and CD133-knockdown FEMX-1 cells revealed an inverse association between CD133 and CD271 expression levels. This is in agreement with our previous observations, obtained by microarray analysis and quantitative reverse transcription-polymerase chain reaction, that small hairpin RNA-mediated downregulation of CD133 expression in human FEMX-I melanoma cells resulted in overexpression of CD271 (14). While CD271 appears to be crucial in maintaining the tumorigenicity and stem-like properties of the majority of melanomas (9,29), previous studies have demonstrated that, in certain cases, CD133 identifies the melanoma stem cell population (14,30). In particular, CD133 expression is crucial for FEMX-I melanoma tumorigenicity and metastatic potential (14). The pro-metastatic role of CD271 is controversial. While in MMs the expression of CD271 is associated with deeply invasive lesions, perineural invasion (31), higher metastatic potential and worse prognosis (10), the majority of invasive and self-renewing phenotypes of medulloblastoma were identified to possess low expression levels of CD271 (32). Although the data presented in the current study suggest that CD271 expression does not define a melanoma stem cell subpopulation in FEMX-I cells, whether the ethanol-induced increase in CD271 expression changes the melanoma stem cell properties of FEMX-I cells will be investigated in a follow-up study. In conclusion, the results of the present study demonstrate an association between ethanol exposure and CD271 expression via the complex NF-κB signaling pathway, which is relevant for the proliferative state of melanoma.

## Figures and Tables

**Table I.** CD271 expression in CD133-knockdown FEMX-I cell lines.

Cell line	CD271^+^ cells, %	Relative fluorescence of CD271^+^ cells
FEMX-I	14±2	1
FEMX-I/SUPER^773/1^	49±3^a^	3.7±0.5^a^
FEMX-V	53±5^a^	2.9±0.4^a^

**Table II.** Flow cytometric analysis of CD271 expression in FEMX-I single-cell clones.

Clone number	CD271^+^, %	Mean fluorescence of CD271^+^ cells^a^
1	4.0	191
2	4.0	66
3	2.8	69
4	20.0	185
5	24.7	170
6	12.5	66
7	13.7	103
8	10.4	163
9	12.5	131
10	15.4	189
11	27.0	225
12	7.4	71
13	10.9	94
14	8.1	69
15	10.2	101
16	3.0	45
17	12.1	105
18	1.4	30
19	0.8	53
20	0.3	31
21	2.8	50
22	1.5	44
23	1.4	43
24	1.5	41

a Arbitrary units.
